# Knowledge and practices of sickle cell disease among healthcare providers in Kinshasa, Democratic Republic of the Congo

**DOI:** 10.4102/phcfm.v16i1.3631

**Published:** 2024-02-15

**Authors:** Ange-Christian M. Ngonde, Jean-Pierre L. Fina, Edu Burgueno, Phillippe N. Lukanu

**Affiliations:** 1 Selembao General Referral Hospital, Kinshasa, The Democratic Republic of the Congo; 2 Department of Family Medicine and Primary Health Care, School of Medicine, Protestant University of Congo, Kinshasa, The Democratic Republic of the Congo; 3 Faculty of Medicine, University of Mwene-Ditu, Kasai, The Democratic Republic of the Congo; 4 Versale Medical Centre, Kinshasa, The Democratic Republic of the Congo

**Keywords:** sickle cell disease, healthcare providers, knowledge, practices, primary health care

## Abstract

**Background:**

In Kinshasa, Democratic Republic of Congo, there is a low evocation of the diagnosis of sickle cell disease (SCD) by first-level healthcare providers (HCPs), most likely because of poor knowledge of the disease.

**Aim:**

To assess the levels of knowledge and practices of SCD and to identify determinants of the practices among primary HCPs.

**Setting:**

Healthcare facilities in Selembao health zone in Kinshasa, Democratic Republic of the Congo.

**Methods:**

A cross-sectional study of HCPs randomly selected through a two-stage sampling design. Data were collected using a pre-tested interviewer-administered questionnaire. Univariate and bivariate analysis were done to describe the levels of knowledge and practices of SCD. Factors associated with better practices on SCD were determined using multiple linear regression. The threshold for statistical significance was *p* ˂ 0.05.

**Results:**

A total of 318 HCPs, which included 80 physicians and 238 nurses, participated in the study. The participants showed different scores on the components of the knowledge. All the participants showed poor practices on SCD. Multiple linear regression retained overall knowledge of SCD as a significant predictor of better practice for physicians. Knowledge of SCD and duration of work experience were significant predictors of better practices among nurses.

**Conclusion:**

The practices of healthcare providers on SCD were far from optimal. These practices were significantly associated with knowledge and experience of healthcare providers.

**Contribution:**

This study highlighted the need for continuing professional education to enhance the management of SCD in the setting.

## Introduction

Sickle cell disease (SCD) is a common genetic disease, the sickle cell trait variant affecting about 300 million people worldwide, with one-third of the cases in sub-Saharan Africa, while the prevalence rates in the United States is 9% among African American people and 0.2% among Caucasian people.^1^ In some areas of sub-Saharan Africa, up to 2% of all children are born with the condition. In broad terms, the prevalence of the sickle-cell trait (healthy carriers who have inherited the mutant gene from only one parent) ranges between 10% and 40% across equatorial Africa and decreases to between 1% and 2% on the north African coast and <1% in Southern Africa.^2^ The disease usually presents between the ages of 6 and 18 months with homozygous carriers of the sickle cell gene having a shortened life expectancy because of multiple complications. The survival of SCD patients depends on the quality of the healthcare systems.^2^ In high-income countries, the median survival of SCD patients is more than 60 years, and childhood mortality is now close to that in the general population. In the lower-income countries of Africa with poor healthcare systems, the under-five mortality of SCD can be as high as 90%.^3,4^

In 2010, the World Health Organization (WHO) recommended a strategy of interventions centred on primary health care and valuing the improvement of healthcare staff skills in the comprehensive management of SCD.^5^ Staff training is one of the cornerstones of the fight against SCD. Activities for management of patients with sickle-cell disease should be based at the primary health care level, with emphasis on programmes that use simple, affordable technology and reach a large proportion of the community, and the personnel involved in medical care will most likely be primary healthcare practitioners with task-oriented basic training in sickle-cell disease.^2^

This study was done in the Democratic Republic of the Congo (DRC), a country with a sickle cell trait AS prevalence of 20% – 30% and a birth rate of 2% of homozygotes.^6^ The state of SCD control is similar to that in Nigeria.^7^ In Kinshasa, a review of data from Makala Referral Hospital, located in Selembao health zone, from 2009 to 2013, noted that sickle cell anaemia was not mentioned in any of the referral notes for children with polytransfusion.^8^ However, given the different causes of anaemia in children and its prevalence in the DRC, one would expect SCD to be suspected in all polytransfused children.^9,10^ The study hypothesised that the lack of suspicion of SCD reflected a lack of knowledge of the disease.

Studies carried out in East and West Africa have revealed a low level of knowledge of the disease among the general population, medical students and some healthcare providers (HCPs).^11,12,13,14^ There are also deficits in various organisational aspects of the control, screening, management and treatment of sickle-cell anaemia, involving several HCPs.^15,16^

The Democratic Republic of the Congo in accordance with the recommendation of the WHO, being a country with a high prevalence of SCD, has set up a national programme to fight against SCD. The vision of this programme is to guarantee access to prevention and treatment services for SCD.^5,17^ No studies on the level of knowledge and practice of HCPs about SCD have been done in DRC. The lack of knowledge and practices would delay the interventions of the HCPs, and consequently the mortality linked to this disease in children under five would still be high. This study aimed to assess the level of knowledge and practices of SCD and to identify determinants of better practice among primary HCPs in Selembao health zone, Kinshasa, DRC.

## Research methods and design

### Study design

This was a cross-sectional study among HCPs in Selembao health Zone, in Kinshasa, DRC.

### Study setting

Selembao health zone is one of 35 health zones in the provincial health division of the city-province of Kinshasa in the DRC.^8^ Selembao health zone covers a population of 478 094 inhabitants in 18 health areas. A health area is a delimited geographical entity, composed of a group of villages in rural areas of villages in rural areas and/or streets in urban areas, established according to socio-demographic affinities, with an average population size of 10 000. The average population served is 10 000, depending on the area (rural or urban).^8^

Each health area is covered by a referral health centre. The zone includes a referral hospital, 18 referral health centres and 140 health centres. The health zone is managed by a management team coordinated by the head of the health zone. The health centres are mainly managed by the nurses while the physicians are working in the referral hospital.

### Study population

The authors recruited HCPs working in the health centres of Selembao health zone. There are three categories of nurses according to their educational level – A2: high school level nurses (4 years in secondary school), A1: graduate level nurses (3 years in college) and A0: university level (2 years post college).

### Sampling size estimation

All the physicians working in the health centres were included in the study. For nurses, the minimum size was calculated using OpenEpi software.^18^ Considering the total number of nurses in the health centres (790), an estimated frequency of knowledge about diagnosis and management of 31%,^19^ at a 95% confidence interval and the design effect of 1 (simple random sample); the minimum sample size was 233. Nurses were selected by simple random sampling at the facility level, with each facility required to give at least two nurses. A total of 80 physicians and 790 nurses were identified in the health centres of the zone.

### Inclusion and exclusion criteria

All HCPs (physicians and nurses) performing consultations in the health centres were eligible for the study. Physicians and nurses specifically assigned to administration services were excluded.

### Sampling procedure

The sampling process was different for physicians and nurses. All the physicians working in the health centres were invited to participate in the study. For the nurses, a two-stage sampling procedure was used. At the first stage, all the health centres of the health zone have been listed and assigned numbers. Then, a simple random sampling using a random number generator was performed. A total of 112 out of 158 facilities were selected. At the second stage, for the selection of HCPs, the nurses were selected also using simple random sampling. All the nurses performing consultations in the selected facilities have been listed and assigned numbers. A random number generator was used to identify the individuals to include in the study.

### Data collection

Data were collected using a questionnaire adapted from a study by Adewoyin et al.^12^ The tool used in the current study had 92 items, divided into five sections: socio-demographic data of the HCPs (3 items), knowledge related to epidemiological data on SCD (9 items), knowledge related to clinical manifestations of SCD (15 items), knowledge related to SCD diagnosis and management (20 items) and practice of SCD diagnosis, crisis management and outpatient follow-up in a health centre (45 items). The data was collected from 15 December 2017 to March 2018.

### Data analysis

After the survey, the data were entered into an Excel file and exported into SPSS v. 20 software for analysis. For each item, the participant was given a score of 1 for a correct answer and 0 for a wrong answer. The total number of correct answers obtained was divided by the total number of items per section to determine the percentage.

Categorisation of the level of knowledge considered the percentage of the total points obtained and the profession for physicians: high level (˃75%), average level (50% – 75%) and low level (˂50%); for nurses: high level (˃65%), average level (50% – 65%) and low level (<50%). For the practice, the authors used the same thresholds and categorised it as better, good and poor practice. The authors determined absolute and relative frequencies of categorical variables, means and standard deviation for continuous normal variables or medians and interquartile range for non-normal variables. Multiple linear regression was performed, separately for nurses and physicians, to identify predictors of better practices. The level of statistical significance is *p* < 0.05.

### Ethical considerations

The study was conducted with the approval of the Ethics and Research Committee of the Protestant University of Congo (Ref N°: CEUPC 0043). Permission to carry out the study was obtained from the Provincial Health District of the city or province of Kinshasa and then approved by the Head of Selembao HZ. Written informed consent was obtained by signature of each participant after explaining the purpose and objectives of the study. Each participant was free to withdraw from the study at any stage without any negative consequences to his or her work. The participants’ responses were analysed as pooled data, and anonymity of the HCPs in the study and confidentiality of their data were always guaranteed.

## Results

A total of 318 HCPs, which included 80 (25.2%) physicians and 238 (74.8%) nurses, took part in the study. Nurses were divided into 118 (49.6%) with high school level (A2), 110 (46.2%) with graduate level (A1) and 10 (4.2%) university level (A0).

Table 1 summarises the socio-demographic characteristics of participants. The mean age of the HCPs, all categories combined, was 37.8 (±9.9) years, with almost half of the HCPs (43.4%) in the 30–39 age group. Males predominated (65.1%) and most of them (31.8%) had more than 10 years of work experience.

**TABLE 1:** Age, sex and experience of healthcare providers working in Selembao health zone.

Variables	Nurses						Physicians (*N* = 80)		All (*N* = 318)	
	A2 (*N* = 118)		A1 (*N* = 110)		A0 (*N* = 10)		*n*	%	*n*	%
	*n*	%	*n*	%	*n*	%				
**Age (years)**										
< 30	18	15.3	33	30.0	0	0.0	5	6.3	56	17.6
30–39	37	31.3	50	45.5	3	30.0	48	60.1	138	43.4
40–49	44	37.2	19	17.3	5	50.0	21	26.3	89	28.0
≥ 50	19	16.1	8	7.3	2	20.0	6	7.5	35	11.0
**Sex**										
Female	47	39.8	55	50.0	3	30.0	6	7.5	111	34.9
Male	71	60.2	55	50.0	7	70.0	74	92.5	207	65.1
**Experience**										
Less than 3 years	20	16.9	22	20.0	0	0.0	24	30.0	66	20.8
3 to 5 years	17	14.4	38	34.5	3	30.0	27	33.8	85	26.7
6 to 10 years	22	18.6	28	25.5	1	10.0	15	18.8	66	20.8
More than 10 years	59	50.0	22	20.0	6	60.0	14	17.5	101	31.8

Physicians had an average level of knowledge about the epidemiology of SCD (65.8%); among nurses, those with a university’s degree (60.0%) and a graduate degree (59.6%) had an average level of knowledge about epidemiology (Table 2).

**TABLE 2:** Distribution of knowledge on sickle cell disease epidemiology among healthcare providers working in Selembao health zone.

Variables	Nurses						Physicians (*N* = 80)		All (*N* = 318)	
	A2 (*N* = 118)		A1 (*N* = 110)		A0 (*N* = 10)		*n*	%	*n*	%
	*n*	%	*n*	%	*n*	%				
Have information on sickle cell disease (SCD)	113	95.8	109	99.1	10	100.0	78	97.5	310	97.5
Continents affected by SCD (Africa, America, Asia, Europe)	0	0.0	2	1.8	0	0.0	3	3.8	5	1.6
The prevalence of the S trait in DRC is more than 10%	30	25.4	31	28.2	6	60.0	33	41.3	100	31.4
The prevalence of SS in DRC is 1% to 2%	15	18.5	16	18.0	1	10.0	17	21.8	49	19.0
Both sexes are affected	80	67.8	79	71.8	9	90.0	52	65.0	220	69.2
University course is the main source of knowledge about SCD	24	20.3	79	71.8	7	70.0	76	95.0	186	58.5
SCD is an inherited disease	101	85.6	101	91.8	9	90.0	76	95.0	287	90.3
SC gene is inherited from two parents	85	72.0	84	76.4	9	90.0	69	86.3	247	77.7
The carrier of the S-trait inherited the gene from one parent	78	66.1	89	80.9	9	90.0	70	87.5	246	77.4

DRC, Democratic Republic of the Congo.

All the HCPs showed a high level of knowledge of the clinical manifestations of SCD: 85.7%, 79.3%, 72.8% and 70.1% for physicians, university level nurses, graduate degree nurses and high school level nurses, respectively (Table 3).

**TABLE 3:** Healthcare provider’s knowledge of the clinical manifestations of sickle cell disease.

Clinical manifestations	Nurses						Physicians (*N* = 80)		All (*N* = 318)	
	A2 (*N* = 118)		A1 (*N* = 110)		A0 (*N* = 10)		*n*	%	*n*	%
	*n*	%	*n*	%	*n*	%				
Anaemia	111	94.1	109	99.1	10	100.0	79	98.8	309	97.2
Bone pain	109	92.4	105	95.5	10	100.0	78	97.5	302	95.0
Swelling of hand/foot	103	87.3	98	89.1	9	90.0	78	97.5	288	90.6
Repeated seizures	101	85.6	101	91.8	9	90.0	73	91.3	284	89.3
Growth and developmental delay	110	93.2	101	91.8	10	100.0	75	93.8	296	93.1
Jaundice	109	92.4	104	94.5	10	100.0	76	95.0	299	94.0
Joint pain	113	95.8	104	94.5	10	100.0	76	95.0	303	95.3
Chronic leg ulcer	49	41.5	51	46.4	7	70.0	55	68.8	162	50.9
Stroke	22	18.6	18	16.4	4	40.0	50	62.5	94	29.6
Priapism	25	21.2	23	20.9	3	30.0	63	78.8	114	35.8
Pulmonary infection	45	38.1	48	43.6	5	50.0	55	68.8	153	48.1
Bone infection	82	69.5	85	77.3	10	100.0	66	82.5	243	76.4
Frequent transfusions	112	94.9	100	90.9	10	100.0	79	98.8	301	94.7
Erythrocytes are short-lived and underlie anaemia	104	88.1	104	94.5	8	80.0	75	93.7	291	91.5
Recurrent infections may suspect SCD	45	38.1	51	46.4	4	40.0	51	63.8	151	47.5

SCD, sickle cell disease.

The proportion of high school and graduate-level nurses with an average level of knowledge about the diagnosis, and management of SCD was 52.8% and 55.9%, respectively (Table 4).

**TABLE 4:** Healthcare provider’s knowledge of diagnosis and management of sickle cell disease.

Variables	Nurses						Physicians (*N* = 80)		All (*N* = 318)	
	A2 (*N* = 118)		A1 (*N* = 110)		A0 (*N* = 10)		*n*	%	*n*	%
	*n*	%	*n*	%	*n*	%				
**Diagnosis**										
diagnosis before 6 months of life	71	60.2	79	71.8	7	70.0	60	75.0	217	68.2
Blood type is not useful	36	30.5	46	41.8	5	50.0	61	76.3	148	46.5
Emmel test is used as a guide	43	36.4	52	47.3	7	70.0	58	72.5	160	50.3
Haemoglobin electrophoresis is used for confirmation	90	76.3	86	78.2	8	80.0	79	98.8	263	82.7
Rapid diagnostic test for SCD is for referral	24	20.3	30	27.3	2	20.0	34	42.5	90	28.3
**Management**										
Treatment of acute crisis										
Abundant hydration	32	27.1	29	26.4	4	40.0	21	26.3	86	27.0
Pain management	87	73.7	80	72.7	6	60.0	75	93.8	248	78.0
Blood transfusion	100	84.7	94	85.5	9	90.0	75	93.8	278	87.4
Oxygenation	69	58.5	65	59.1	7	70.0	67	83.8	208	65.4
Maintenance treatment										
Folic acid	72	61.0	64	58.2	7	70.0	64	80.0	207	65.1
Breastfeeding can be encouraged	55	46.6	54	49.1	7	70.0	40	50.0	156	49.1
Deworming	61	51.7	53	48.2	8	80.0	47	58.8	169	53.1
Oral penicillin	43	36.4	32	29.1	6	60.0	42	52.5	123	38.7
Vaccination (routine and extends to other germs)	54	45.8	62	56.4	8	80.0	58	72.5	182	57.2
Therapeutic education about the disease and its consequences	75	63.6	74	67.3	9	90.0	66	82.5	224	70.4
Hydration	25	3.4	27	1.8	5	50.0	24	11.3	81	25.5
Hydroxyurea to reduce frequency of attacks	15	12.7	17	15.5	4	40.0	38	47.5	74	23.3
Prevention										
Expand community screening to adolescents	100	84.7	93	84.5	9	90.0	73	91.3	275	86.5
Offer premarital screening	100	84.7	103	93.6	10	100.0	77	96.3	290	91.2
Perform provider-initiated screening	96	81.4	90	81.8	9	90.0	69	86.3	264	83.0

SCD, sickle cell disease.

Table 5 details the practices of the healthcare provides on SCD: use of screening tests, description of symptoms or signs allowing to evoke the diagnosis of SCD, the performance of diagnostic tests and the management. Only physicians had good practices on SCD (50.1%); all nurses regardless of educational level had low practices with 42.6%, 44.2% and 40.7% for university level nurses, graduate level nurses and high school nurses, respectively (Table 5).

**TABLE 5:** Healthcare provider’s practice related to diagnosis, crisis management and outpatient follow-up of sickle cell patients.

Variables	Nurses						Physicians (*N* = 80)		All (*N* = 318)	
	A2 (*N* = 118)		A1 (*N* = 110)		A0 (*N* = 10)		*n*	%	*n*	%
	*n*	%	*n*	%	*n*	%				
**Screening or diagnosis**										
Haemoglobin profile	52	44.1	54	49.1	9	90.0	54	67.5	169	53.1
Practitioner diagnosed at least one case of SCD	78	66.1	73	66.4	6	60.0	72	90.0	229	72.0
**Symptoms or signs evoking diagnosis**										
Fever	60	50.8	63	57.3	4	40.0	49	61.3	176	55.3
Pain	89	75.4	83	75.5	6	60.0	76	95.0	254	79.9
Cutaneous-mucosal pallor	86	72.9	75	68.2	4	40.0	73	91.3	238	74.8
Notion of previous transfusion	82	69.5	78	70.9	5	50.0	74	92.5	239	75.2
Jaundice	87	73.7	75	68.2	6	60.0	62	77.5	230	72.3
Swelling of hands and feet in infants	69	58.5	63	57.3	4	40.0	65	81.3	201	63.2
Notion of multiple deaths in the immediate family	58	49.2	58	52.7	4	40.0	40	50.0	160	50.3
**Assessment of pain**										
Subjective (based on patient complaint)	105	89.0	97	88.2	9	90.0	73	94.3	284	89.3
Using a scale	11	9.3	13	11.8	0	0.0	18	22.5	42	13.2
**Use of usual analgesics**										
Empirically	62	52.5	63	57.3	5	50.0	42	52.5	172	54.1
According to WHO guidelines	56	47.5	59	53.6	5	50.0	44	55.0	164	51.6
**Analgesics used**										
Paracetamol	57	48.3	40	36.4	5	50.0	22	27.5	124	39.0
Paracetamol and codéine	17	14.4	15	13.6	1	10.0	21	26.3	54	17.0
NSAIDs (diclofenac, ibuprofen…)	38	32.2	38	34.5	3	30.0	23	28.8	102	32.1
Tramadol	73	61.9	56	50.9	6	60.0	39	48.8	174	54.7
Morphine	1	0.8	0	0.0	0	0.0	0	0.0	1	0.3
**Other treatments**										
Transfusion	9	7.6	14	12.7	1	10.0	12	15.0	36	11.3
Hydration with saline	12	10.2	15	13.6	2	20.0	14	17.5	43	13.5
Oxygen	35	29.7	35	31.8	4	40.0	39	48.8	113	35.5
Hydroxyurea	10	8.5	5	4.5	2	20.0	12	15.0	29	9.1
**Outpatient follow-up**										
Provider has ever followed a SCD patient	40	33.9	38	34.5	5	50.0	32	40.0	115	36.2
SCD follow-up may be once a month	27	22.9	27	24.5	4	40.0	22	27.0	80	25.2
**Laboratory tests**										
Haemoglobin. white blood cell count. complete blood count	103	87.3	97	88.2	8	80.0	71	88.8	279	87.7
Sedimentation rate	15	12.7	7	6.4	1	10.0	4	5.0	27	8.5
C-reactive protein	8	6.8	16	14.5	0	0.0	4	5.0	28	8.8
Lactate dehydrogenase	20	16.9	35	31.8	2	20.0	20	25.0	77	24.2
Urea and creatinine	38	32.2	19	17.3	2	20.0	53	66.3	112	35.2
Transaminases	24	20.3	34	30.9	4	40.0	41	51.3	103	32.4
Total/indirect bilirubin	38	32.2	26	23.6	4	40.0	52	65.3	120	37.7
10-reagent urine dipstick	19	16.1	44	40.0	2	20.0	21	26.3	86	27.0
Conventional radiography	37	31.4	61	55.5	4	40.0	38	47.5	140	44.0
Abdominal ultrasound	46	39.0	60	54.5	4	40.0	54	67.5	164	51.6
Cardiac ultrasound	47	39.8	30	27.3	4	40.0	45	56.3	126	39.6
Transcranial doppler ultrasound	24	20.3	46	41.8	4	40.0	29	36.3	103	32.4
Electrocardiogram	41	34.7	28	25.5	2	20.0	40	50.0	111	34.9
CT scan	34	28.8	21	19.1	1	10.0	25	33.8	81	25.5
**Reason for transfer**										
Medical logistic and financial	80	67.5	72	65.5	7	70.0	48	60.0	207	65.1
Limited clinical skills of the centre	92	78.0	78	70.9	7	70.0	48	60.0	225	70.8
**Medical reasons for referral**										
Pain resistant to usual analgesics	98	83.1	88	80.0	6	60.0	46	57.5	238	74.8
Persistent infection	101	85.6	100	90.9	5	50.0	53	66.3	259	81.4
Decompensated anaemia	97	82.2	92	83.6	8	80.0	67	83.8	264	83.0
**Reference-counter-reference**										
Provider develops referral score	90	76.3	88	80.0	8	80.0	65	81.3	251	78.9
Provider receive counter-reference score	18	15.3	11	10.0	0	0.0	3	3.8	32	10.1

SCD, sickle cell disease; WHO, World Health Organizations; CT, computed tomography; NSAIDs, Non-steroidal anti-inflammatory drugs.

For physicians, multiple linear regression was used to test whether age, duration of occupation and knowledge of the disease predicted sickle cell practice. The overall regression was statistically significant (*R*^2^ = [0.3360], *F*[7.62] = [4.48], *p* = [0.0004]). It was found that SCD knowledge predicted SCD practice (ß = [0.4592492], *p* = [0.000]).

For nurses, multiple linear regression was used to test whether age, title, duration of occupation and knowledge of the disease predicted sickle cell practice. The overall regression was statistically significant (*R*^2^ = [0.3571], *F*[30.186] = [3.44], *p* = [0.0000]). Sickle cell disease knowledge and duration of work experience were found to predict SCD practice.

## Discussion

The results of this study conducted to assess the knowledge and practices of primary HCPs on SCD showed an average level of knowledge about the diagnosis and management. Most participants were male, and a significant number had more than 10 years of work experience. The physicians showed good practice while all the nurses had a poor practice of SCD. Better practice on SCD practice was associated with knowledge among physicians and with knowledge and time in practice among nurses.

The predominance of male health care providers in the study sample highlights the existence of gender inequality within the medical workforce in the designated health zone of Kinshasa, DRC. Elsewhere, there is a tendency for female-predominant PHC organisations’ healthcare.^20^ It was found that women providers have a more patient-centred communication and are more likely to meet quality performance measures.^21^ Addressing gender disparities could enhance provision and ensure optimal patient outcomes.

The HCPs show either average level (physicians, university and graduate level nurses) or poor level of knowledge on the epidemiology of SCD. It is essential for the HCPs to have a good knowledge on the epidemiology of sickle cell disease to implement effective preventive measures and tailor interventions to the needs of the population. The average level of knowledge indicates a potential gap and underscores the need for enhanced training and educational initiatives. Another area for training would be knowledge on clinical diagnosis and management where the health providers also showed average knowledge. And the situation is not limited to Kinshasa; a study in Kindu in the Eastern part of the DRC found poorer knowledge among HCPs with 48.8% and 26.6% having awareness of Emmel test and haemoglobin electrophoresis, respectively.^22^ A study in Tanzania showed that only 25.1% had good knowledge on SCD.^23^ Encouragingly, participants demonstrated a high level of knowledge about the clinical manifestations of SCD. This is a positive indicator, as recognition of the signs and symptoms of SCD is essential for timely diagnosis and intervention.

A slightly more than half of the physicians reported having performed activities related to the diagnosis, management of crises and ambulatory follow-up of sickle cell patients. For the nurses, fewer than half the participants reported having performed these activities. Gomes and colleagues found a better practice than in the current study (65.5%), ranging from 40.4% to 87.23%.^22^ It is interesting to note that the authors considered physicians and nurses without distinction and addressed the knowledge of management only on children. It is noteworthy that the study by Gomes and colleagues assessed HCPs as a collective group, without distinguishing between physicians and nurses, and focused solely on the knowledge of managing paediatric populations. This may explain the observed higher level of practice, as the assessment of physicians may compensate for the potentially lower level of nurses, and the surveyed medical staff exclusively treated paediatric populations, which are likely to encounter a higher frequency of SCD cases. In contrast, the current study conducted separate assessments for nurses and physicians and included providers who were not exclusively dedicated to treating children.

This analysis using multivariate linear regression found that for physicians, there was a significant relationship between their knowledge of SCD and their practices in managing the condition. For nurses, both their knowledge of SCD and the duration of their work experience in the profession were significant predictors of their practice in managing the disease. This distinction between the predictors for physicians and nurses may be attributed to the compensatory effect of extensive professional experience for nurses, which may mitigate the impact of a lower level of academic knowledge. Essentially, nurses may enhance their understanding and management of SCD through accumulated field experience, thereby improving their practice in managing the condition.^25,26^ Nevertheless, for this knowledge to be translated in practice, health centres must have the necessary equipment for diagnosing SCD. A multisite study in the DRC showed that 85% of the participants, from which the most were physicians, had no access to diagnostic tools of sickle disease.^26^ The study in Tanzania also showed that regional-level hospitals lacked diagnostic tests and hydroxyurea.^23^ The findings of the current study are corroborated by studies that have linked practices on sickle cell disease with either the knowledge of the disease^16^ or provider qualification.^24^

### Strengths and limitations

To the best of our knowledge, this is the first study in the sub-Saharan Africa to deal with the factors associated with better practice on SCD. The study used a detailed questionnaire to identify areas for possible improvement in terms of knowledge and practice.

However, some limitations should be considered. Firstly, the study was conducted in a specific health zone in Kinshasa, DRC, which might limit the generalisability of the findings to other regions or countries with different healthcare systems and demographics. Secondly, the reliance on self-reported data from HCPs may introduce response bias. Thirdly, because of its cross-sectional design, it is not possible to conclude on definitive relationship between factors identified for better practice on SCD.

Nonetheless, this study sheds light on the current state of knowledge and practices among HCPs in the health zone regarding SCD. Addressing the factors identified as associated with practice could enhance the quality of SCD care.

## Conclusion

This study found that better practices on SCD were associated with either the level of knowledge on the disease (for all the HCPs) or with the duration of work experience for nurses. Raising the level of knowledge and improving the practices of the HCPs through continuing professional education might be beneficial in implementing opportunistic prevention of SCD in the setting. Taking into account the prevalence of SCD in the study setting, health centres must be equipped with basic equipment to ensure the diagnosis of the disease.
